# Isolation of Heartland Virus from Lone Star Ticks, Georgia, USA, 2019

**DOI:** 10.3201/eid2804.211540

**Published:** 2022-04

**Authors:** Yamila Romer, Kayla Adcock, Zhuoran Wei, Daniel G. Mead, Oscar Kirstein, Steph Bellman, Anne Piantadosi, Uriel Kitron, Gonzalo M. Vazquez-Prokopec

**Affiliations:** Emory University, Atlanta, Georgia, USA (Y. Romer, Z. Wei, O. Kirstein, S. Bellman, U. Kitron, G.M. Vazquez-Prokopec);; University of Georgia College of Veterinary Medicine, Athens, Georgia, USA (K. Adcock, D.G. Mead);; Emory University School of Medicine, Atlanta (A. Piantadosi)

**Keywords:** Heartland virus, HRTV, Bourbon virus, BRBV, viruses, Amblyomma americanum, ticks, vector-borne infections, zoonoses, Georgia, United States

## Abstract

Report of a human death and exposure of white-tailed deer to Heartland virus (HRTV) in Georgia, USA, prompted the sampling of questing ticks during 2018–2019 in 26 sites near where seropositive deer were captured and the residence of the human case-patient. We processed 9,294 *Amblyomma americanum* ticks in pools by virus isolation in Vero E6 cells and reverse transcription PCR. Positive pools underwent whole-genome sequencing. Three pools were positive for HRTV (minimum infection rate 0.46/1,000 ticks) and none for Bourbon virus. Cell cultures confirmed HRTV presence in 2 pools. Genome sequencing, achieved for the 3 HRTV isolates, showed high similarity among samples but marked differences with previously sequenced HRTV isolates. The isolation and genomic characterization of HRTV from *A. americanum* ticks in Georgia confirm virus presence in the state. Clinicians and public health professionals should be aware of this emerging tickborne pathogen.

In 2009, a novel phlebovirus, Heartland virus (HRTV), was identified as the cause of illness in 2 severely ill patients from Missouri, USA (*1*), after exposure to lone star ticks (*Amblyomma americanum*). HRTV, an RNA virus recently reclassified as belonging to the family *Phenuiviridae* and genus *Bandavirus* (*2*), is closely related to severe fever with thrombocytopenia syndrome virus (SFTSV), which is transmitted mainly by longhorned ticks (*Haemaphysalis longicornis*) and causes a hemorrhagic fever in Southeast and central Asia (*3*).

The ecology and natural history of HRTV remain largely unknown (*4*). The virus was isolated from the initial index cases in Missouri and from ticks collected at nearby sites (*1*,*5*). Viral RNA has been detected by molecular tools in immature and mature stages of *A. americanum* ticks from Missouri (*5*,*6*), Alabama (*7*), Illinois (*8*), Kansas (*9*), and New York (*10*). Antibodies reactive to HRTV have been identified in various wildlife species (*11*–*13*) that match the geographic distribution of *A. americanum* ticks in the United States (*14*), even in those areas where the presence of the tick is scarce. Nonetheless, viremia in a vertebrate species has not been detected (*11*,*13*) and attempts to induce viremia in experimental vertebrate hosts have been unsuccessful (*15*,*16*).

Since HRTV was identified in 2009, ≈40 additional human cases of HRTV disease have been identified in Missouri, Kansas, Oklahoma, Arkansas, Iowa, Illinois, Tennessee, Indiana, Georgia, and South Carolina (*8*,*17*–*20*). Most of these cases were reported in persons who had underlying conditions, and their illnesses were predominately severe or fatal (*4*,*21*). However, seroprevalence studies in wildlife suggest a broader range of distribution of HRTV than those states from which cases of human disease have been reported (*13*).

A second novel tickborne arbovirus, Bourbon virus (BRBV), was isolated from a fatal human case (*22*) and from field-collected arthropods (*9*) in Bourbon County, Kansas, USA, during 2014. BRBV has a negative-sense RNA genome of 6 segments and represents the only member of the genus *Thogotovirus* that causes human disease in the Western Hemisphere; a limited number of persons have been infected in the midwestern and southern United States. This virus was also linked to *A. americanum* ticks in Missouri (*23*) at a lower infection rate than that for HRTV infection. Wildlife seroprevalence studies suggest a wide distribution of BRBV in the southeastern United States (*24*), but human disease remains a rare event.

Although *A. americanum* ticks are widely distributed throughout the southeastern United States, only 1 study, in Alabama, has conclusively identified HRTV in lone star ticks in this region (*7*). In Georgia, there is serologic evidence of HRTV infection in white-tailed deer (*Odocoileus virginianus*) dating back to 2001. A single hu­man infection from 2005 was confirmed in 2015 (https://www.cdc.gov/heartland-virus/statistics/index.html). Because *A. americanum* ticks represent ticks most frequently associated with human bites in Georgia (*25*), we examined *A. americanum* ticks for arboviruses, and specifically for HRTV and BRBV, in a select area in Georgia to better assess the risk for human disease in the region and to increase knowledge of the ecology and genomics of these emerging human pathogens.

## Materials and Methods

### Study Area

**Figure 1:**
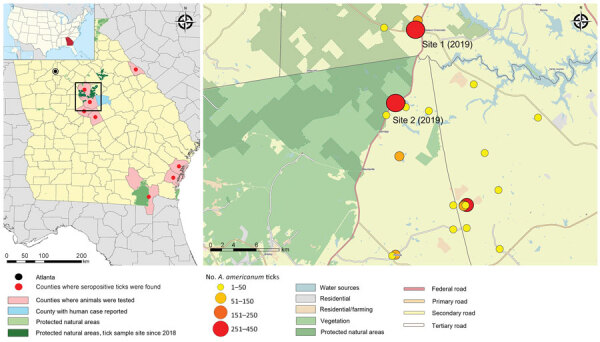
Study area for investigation of Heartland virus ecoepidemiology, Georgia, USA, 2019. Map on the left shows locations of seropositive white-tailed deer and 1 human case; inset shows location of study area in Georgia. Map on the right shows *Amblyomma americanum* tick collection sites during 2018 and the 2 sites during 2019. Circle sizes and colors indicate number of ticks collected.

The study area in Georgia was a 64 km^2^ rural landscape located ≈130 km southeast of Atlanta and situated adjacent to the Piedmont National Wildlife Refuge (latitude 33.117934, longitude −83.413621). This area includes parts of Jones, Baldwin, and Putnam Counties in central Georgia and had a cumulative population of 93,180 inhabitants as of 2010 (US Census Bureau, https://www.census.gov/2010census/data). The area is part of the southern Piedmont ecoregion, comprising predominantly deciduous woodlands (Figure 1). The climate is humid subtropical, has a mean annual high temperature of 31°C and low temperature of 13°C, and has mean annual precipitation of 115 cm.

We selected this study area on the basis of data from a seroprevalence evaluation of white-tailed deer for antibodies to HRTV (*12*) and from its proximity to the only reported case of human HRTV infection (https://www.cdc.gov/heartland-virus/statistics/index.html) (Figure 1). During 2018, we collected samples from 26 different sites around the area to identify *A. americanum* tick productive sites. During 2019, we focused our efforts in the 2 sites that yielded the highest collections during 2018. All sites were favorable, on a visual inspection, for development of the different stages of *A. americanum* ticks (presence of deciduous forest, open access to diverse fauna, and grass cover). The 2 locations sampled during 2019 were a vacant lot (33.155975, −83.450516) and a private property (33.201552, −83.439257) (the collection activity was approved by their owners), which were 5.41 km apart (Figure 1).

### Tick Collection Strategy and Entomologic Identification

We collected ticks approximately each week during April‒October in 2018 and 2019. We collected host-seeking adults and nymphal ticks by using flannel flags and transported them alive to the laboratory, where we identified them microscopically on a chilled table for sex, species, and life stage by using taxonomic keys (*26*,*27*).

### Tick Processing

We surface disinfected live ticks by sequential immersion for 5 min in cold solutions of 70% ethanol, 10.5% sodium hypochlorite, and 3% hydrogen peroxide, and then rinsed them in distilled water (*28*). We pooled live specimens by species, collection site, and stage in groups of <5 adults and <25 nymphs. We added 1 mL of BA-1 diluent (1× medium 199 with Hanks balanced salt solution, 0.05 mole/L Tris buffer [pH 7.6], 1% bovine serum albumin, 0.35 g sodium bicarbonate/L, 100 μg/L streptomycin, 1 μg/mL amphotericin B) to each pool (*29*) before grinding the pools thoroughly by using a 7-mL glass TenBroeck grinder (Fisher Scientific, https://www.fishersci.com) with alundum Bedding material (Fisher Scientific) as an abrasive. We transferred each homogenate to a sterile 2-mL cryotube and stored the tubes at −80°C for future analysis.

### Molecular Detection and Cell Culture Isolation

We thawed tick homogenates and centrifuged them at 14,000 rpm for 10 min to clarify before proceeding. We extracted total RNA from each homogenate by using a QIAmp RNA Extraction Kit (QIAGEN, https://www.qiagen.com). We performed a quantitative real-time PCR with a final reaction volume of 25 μL and 1 μL of template by using a QuantiTect Probe PCR Kit (QIAGEN), with primer-probe set 1, which was designed for the small segment of the HRTV genome, as described by Savage et al. (*6*) under the following cycling conditions: 50°C for 30 min; 95°C for 10 min; and 45 cycles with 1 cycle consisting of 95°C for 15 s and 60°C for 1 min. We performed BRBV screening in a separate quantitative real-time PCR by using the primer-probe set NP1, as described (*9*).

We attempted viral isolation in Vero E6 cells with each tick homogenate pool that yielded a positive result by real-time PCR. We inoculated 100 μL of an undiluted sample into a 12-well tissue culture plate and incubated it at 37°C. We monitored cells daily for cytopathic effect; when noted, we removed 140 μL of medium and processed for RNA extraction. We subcultured and monitored cultures with no demonstrable cytopathic effect by day 11 daily for an additional 7 days for cytopathic effect; if no cytopathic effect was noted, we performed a final subculture and monitored for an additional 11 days. We tested RNA extracted from cell culture supernatants (1 μL) by using a real-time PCR specific for HRTV to confirm viral infection.

### HRTV Genome Sequencing and Analysis

Extracted RNA underwent heat-labile, dsDNase treatment (ArcticZymes, https://arcticzymes.com), random primer cDNA synthesis (New England Biolabs, https://www.neb.com), Nextera XT tagmentation (Illumina, https://www.illumina.com), and sequencing (Illumina). We obtained 3.9–5.6 million reads/sample (Appendix Table 1). We performed reference-based assembly by using viral-ngs version 2.1.19 (https://viral-ngs.readthedocs.io/en/latest), using GenBank reference sequences NC_024496 for the small gene segment, NC_024494 for the medium segment, and NC_024495 for the large segment. A minimum of 3 reads was required to call a consensus nucleotide. We aligned the consensus HRTV genomes from each sample with all available reference sequences by using Geneious Prime version 2021.1.1 (https://www.geneious.com). We constructed maximum-likelihood phylogenetic trees by using PhyML (*30*) and visualized trees by using FigTree (http://tree.bio.ed.ac.uk/software/figtree). Assembled HRTV genomes are available in GenBank (accession nos. MZ617368‒76).

**Table 1:** Collected tick species and life cycle stages, Georgia, USA, 2018 and 2019

Tick species and stage	2018	2019	2019, site 1	2019, site 2
*Amblyomma americanum*				
Adult	646	1,530	790	740
Nymph	2,265	4,853	2,844	2,009
Total	2,911	6,383	3,634	2,749
Pools	272	677	339	338
*Amblyomma maculatum*				
Adult	30	10	6	4
Pools	6	9	5	4
*Dermacentor variabilis*				
Adult	14	74	57	17
Pools	3	37	27	10
*Ixodes scapularis*				
Adult	5	3	3	0
Pools	2	2	2	0

### Infection Prevalence Estimation

We estimated the HRTV infection rate in ticks by study site and overall. We calculated this rate by using the minimum infection rate (MIR) per 1,000 ticks (*31*).

## Results

We collected 2,960 ticks during 10 collections during April‒October 2018, comprising 2,265 nymphs and 646 adults of *A. americanum* ticks, 30 adults of *A. maculatum* ticks, 14 adults of *Dermacentor variabilis* ticks, and 5 adults of *Ixodes scapularis* ticks. We collected 6,470 ticks during 10 collections during April‒October 2019, comprising 4,853 nymphs and 1,530 adults of *A. americanum* ticks, 74 adults of *D. variabilis* ticks, 3 adults of *I. scapularis* ticks, and 10 adults of *A. maculatum* ticks. We sorted specimens into 283 pools during 2018 and 677 pools during 2019, by species, stage, sex, and collection site (Table 1). We detected 3 *A. americanum* tick HRTV PCR‒positive pools: 1 pool of 5 females (pool 23, cycle threshold [C_t_] 25.9), 1 pool of 5 males (pool 504, C_t_ 29.9), and 1 pool of 25 nymphs (pool 26, C_t_ 25.6). Two positive pools originated from site 1 and the third pool from site 2 (Figure 1; Table 2).

**Table 2:** HRTV-positive *Amblyomma americanum* tick pools from Putnam and Jones Counties, Georgia, 2019*

No. pools	Collection date	Site	No. specimens in pool	HRTV real-time PCR result for homogenate	Vero E6 cells							
					P1			P2			P3	
					CPE	PCR		CPE	PCR		CPE	PCR
23	Apr 28	1	5	+	‒	‒		‒	‒		‒	‒
26	Apr 28	1	25	+	+	+		‒	+		‒	‒
504	Jun 14	2	5	+	‒	+		‒	+		‒	+

*CPE, cytopathic effect; HRTV, Heartland virus; P1, primary passage; P2, subculture 2; P3, subculture 3. ‒ negative; +, positive.

We performed virus isolation in Vero E6 cells on aliquots of each real-time PCR‒positive homogenate. Pool 26 showed cytopathic effect of Vero E6 cells on day 3 and was passaged again on day 4. Pools 23 and 504 did not show cytopathic effect after the primary passage (P1) or 2 successive subcultures (P2 and P3). However, the supernatant was positive by real-time PCR for P1 of pools 26 and 504, P2 of pools 26 and 504, and P3 of pool 504. C_t_ values for pool 504 were increasing between P1 (C_t_ 26), P2 (C_t_ 20), and P3 (C_t_ 16); C_t_ was 22 for the positive controls. BRBV real-time PCR results were negative for all samples from 2018 and 2019. The MIR of *A. americanum* ticks from site 1 was 0.35/1,000 ticks for nymphs and 1.26/1,000 ticks for adults during 2019; the MIR for adults of *A. americanum* ticks from site 2 was 1.35/1,000 ticks during 2019. Overall, the MIR for the study area was 0.46/1,000 ticks during 2019.

**Figure 2:**
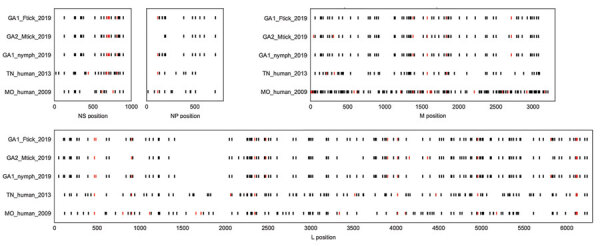
Single-nucleotide polymorphisms in the coding regions of the NS, NP, M, and L open reading frames of Heartland virus collected from ticks and humans in multiple US states. Sequences from this study and other complete Heartland virus sequences from GenBank were compared with reference sequences NC_024496.1, NC_024495.1, and NC_024494.1 (obtained from a patient in Missouri during 2009). Black bars indicate a synonymous mutation, and red bars indicate a nonsynonymous mutation. Plots show considerable variability at the nucleotide level, although the Georgia tick share many single-nucleotide polymorphisms when compared with the reference. GA1_Ftick_2019 corresponds to pool 23, GA1_nymph_2019 corresponds to pool 26, GA2_Mtick_2019 corresponds to pool 504, TN_human_2013 corresponds to a human case from Tennessee (accession nos. KJ740148.1, KJ740147.1, KJ740146.1) and MO_human_2009 corresponds to a human case from Missouri (accession nos. JX005847.1, JX005845.1, JX005843.1). L, large segment; M, matrix protein; NP, nucleoprotein; NS, nonstructural protein.

We sequenced HRTV genomes from each of the 3 positive tick homogenates, which yielded nearly complete HRTV sequences (95.7%–100% coverage for all 3 genome segments) from pools 23 and 26 and only a partial sequence (85.1%, 99.5%, and 88.8% coverage for the small, medium, and large segments) from pool 504 (Appendix Table 1). Sequences from pools 23 and 26, which were obtained at site 1 during 2019, were 99.8%–100% identical to each another in all 4 viral open reading frames (Figure 1; Appendix Figure, Table 1). The sequences were more closely related to the sequence from pool 504 than they were to the 5 previously published HRTV genomes, which had been obtained from 2 patients in Missouri during 2009, one patient in Tennessee during 2013, and 2 ticks from New York during in 2018 (*10*) (Appendix Table 2, Figure 1). Overall, there were a greater number of synonymous changes than nonsynonymous changes (Figure 2), and there were 6 amino acid positions at which all 3 samples from ticks differed from all 3 samples from humans: nonstructural protein positions Q233R, G236E, and R238C; glycoprotein position K903R; and polymerase positions N1300S and A1937S.

## Discussion

We provide evidence of locally infected *A. americanum* ticks with HRTV in Georgia. In the southeastern United States, *A. americanum* ticks are most frequently associated with human bites (*32*). Rapid range expansion and increasing prevalence of the lone star tick (*14*), coupled with anthropologic factors that place humans in tick-infested habitats (*33*), are increasing the human risks for tickborne pathogen spillover. Despite the apparent widespread distribution of HRTV in the southeastern United States, as shown by seroprevalence studies on wildlife (*11*–*13*), detecting HRTV in *A. americanum* ticks has been challenging because of its low infection rate and the typically aggregated nature of arbovirus infections in ticks (*34**,**35*). By focusing sampling efforts in an area with reported exposure to HRTV in wildlife and humans and testing for infection in thousands of ticks from multiple sites and physiologic stages, we confirmed the presence of HRTV in Georgia.

Our sampling effort focused on 2 areas that had high tick density and occurred during the peak of seasonal tick activity to ensure sufficient sample size to enable virus detection. Positive samples were detected in mid-April at site 1 and in mid-June at site 2. Adult and nymph specimens of *A. americanum* ticks were found to be infected, consistent with previous reports that showed that both mature and immature stages of the ticks are infected and competent vectors (*5*,*6*). The finding of infected adults and nymphs in April, early during *A. americanum* tick seasonality, also suggests that HRTV might be overwintering in these ticks. Estimates of MIR are highly variable between regions, years, and season, but are characteristically low, similar to infection prevalence of the closely related tickborne SFTSV in Asia (*36*). Our calculated infection rate is among the lowest in the spectrum reported in other states, which coincides with the rarity of the occurrence of clinical cases, although the possibility of underdiagnoses caused by low awareness of the disease must also be considered; studies from Missouri showed an overall MIR of 1.7/1,000 ticks (*6*), and others from Illinois reported 9.46/1,000 ticks (*8*). A recent study in New York reported MIRs <1.1% (*10*).

Two of 3 samples that had homogenates positive for HRTV by PCR were successfully isolated in cell lines. The complete lysis of the monolayer was observed only in the first culture (P1) of 1 pool (pool 26). The virus was successfully detected in a subculture (P2) but could not be maintained after subsequent passages. In another pool (pool 504), although no cytopathic effect was seen, the virus was detected in the supernatant of 3 consecutive cell line passages in increasing quantities, suggesting successful viral amplification. One positive homogenate was not detected in culture, which was consistent with the slightly decreased sensitivity of virus isolation compared with molecular methods, although it could also correspond to the presence of nonviable virus. Our quantification of C_t_ values throughout passages confirmed viral RNA replication without the need to conduct assays such as immunofluorescent antibody assay or Western blotting. Whereas some investigators might see this result as a limitation, multiple studies confirm the validity of C_t_ values for quantifying virus replication (e.g., *37*,*38*). We emphasize the need for attempting viral isolation, which provides a unique source for phenotypic characterization and pathogenesis studies. Detection of HRTV in ticks to confirm virus circulation in an area has the limitation of low sensitivity because of a low prevalence of infection, as reported in other studies showing low infection rate in ticks (*6*,*8*,*10*). Use of complementary tools, such as serosurveys for vertebrate hosts, could enhance the efficiency in detecting risk areas for human exposure.

Analysis of HRTV genome sequences showed a relatively high degree of conservation between the 3 samples in this study, which were obtained within 2 months of each another and from sites 5 km apart. Because sequencing was performed from pooled tick samples, it is possible that each consensus sequence reflects >1 infected tick. The HRTV genome sequences generated in this study were 2%–5% different from the only 3 other available complete HRTV genome sequences sampled from humans across different states over the preceding decade. This finding reflects a degree of genetic diversity similar to that described for the related *Bandavirus* SFTSV in South Korea (*39*). However, further work is needed to characterize the full spectrum of diversity of HRTV in the United States, and in particular to assess whether there are viral genetic features associated with human infection. Our results demonstrate the feasibility of sequencing complete HRTV genomes directly from tick samples, which enables molecular characterization, a critical step in understanding the diversity, evolution, and pathogenesis of the virus.

Our findings confirm the ongoing circulation of HRTV in Georgia. Major knowledge gaps in the biology and epidemiology of HRTV require further efforts to understand which vertebrates or secondary tick species might play a role in the maintenance of the virus in nature. For instance, the presence and ongoing range expansion of the Asian longhorned tick, *H. longicornis*, in the United States (*40*) could lead to major changes in the transmission ecology of HRTV in areas where this species overlaps with *A. americanum*. Therefore, assessing the current and future risk for HRTV transmission and spillover becomes relevant for disease ecologists and public health practitioners. In the immediate term, knowledge about the presence of HRTV in local ticks would enable improved preventive strategies to mitigate human exposure to ticks, as well as alerting physicians about the presence of this emerging tickborne virus.

Appendix**.** Additional information on isolation of Heartland virus from lone star ticks, Georgia¸ USA, 2019.

## References

[1] McMullan LK, Folk SM, Kelly AJ, MacNeil A, Goldsmith CS, Metcalfe MG, et al. A new phlebovirus associated with severe febrile illness in Missouri. N Engl J Med. 2012;367:834–41. 10.1056/NEJMoa1203378 22931317

[2] Matsuno K, Weisend C, Travassos da Rosa AP, Anzick SL, Dahlstrom E, Porcella SF, et al. Characterization of the Bhanja serogroup viruses (Bunyaviridae): a novel species of the genus Phlebovirus and its relationship with other emerging tick-borne phleboviruses. J Virol. 2013;87:3719–28. 10.1128/JVI.02845-12 23325688 PMC3624231

[3] Liu Q, He B, Huang SY, Wei F, Zhu XQ. Severe fever with thrombocytopenia syndrome, an emerging tick-borne zoonosis. Lancet Infect Dis. 2014;14:763–72. 10.1016/S1473-3099(14)70718-2 24837566

[4] Brault AC, Savage HM, Duggal NK, Eisen RJ, Staples JE. Heartland virus epidemiology, vector association, and disease potential. Viruses. 2018;10:498. 10.3390/v10090498 30223439 PMC6164824

[5] Savage HM, Godsey MS, Lambert A, Panella NA, Burkhalter KL, Harmon JR, et al. First detection of heartland virus (Bunyaviridae: Phlebovirus) from field collected arthropods. Am J Trop Med Hyg. 2013;89:445–52. 10.4269/ajtmh.13-0209 23878186 PMC3771279

[6] Savage HM, Godsey MS Jr, Panella NA, Burkhalter KL, Ashley DC, Lash RR, et al. Surveillance for Heartland virus (Bunyaviridae: Phlebovirus) in Missouri during 2013: first detection of virus in adults of *Amblyomma americanum* (Acari: Ixodidae). J Med Entomol. 2016;53:607–12. 10.1093/jme/tjw028 27032416

[7] Newman BC, Sutton WB, Moncayo AC, Hughes HR, Taheri A, Moore TC, et al. Heartland virus in Lone Star ticks, Alabama, USA. Emerg Infect Dis. 2020;26:1954–6. 10.3201/eid2608.200494 32687045 PMC7392462

[8] Tuten HC, Burkhalter KL, Noel KR, Hernandez EJ, Yates S, Wojnowski K, et al. Heartland virus in humans and ticks, Illinois, USA, 2018‒2019. Emerg Infect Dis. 2020;26:1548–52. 10.3201/eid2607.200110 32568061 PMC7323525

[9] Savage HM, Godsey MS Jr, Tatman J, Burkhalter KL, Hamm A, Panella NA, et al. Surveillance for Heartland and Bourbon Viruses in Eastern Kansas, June 2016. J Med Entomol. 2018;55:1613–6. 10.1093/jme/tjy103 29947778

[10] Dupuis AP II, Prusinski MA, O’Connor C, Maffei JG, Ngo KA, Koetzner CA, et al. Heartland Virus Transmission, Suffolk County, New York, USA. Emerg Infect Dis. 2021;27:3128–32. 10.3201/eid2712.211426 34648421 PMC8632170

[11] Bosco-Lauth AM, Panella NA, Root JJ, Gidlewski T, Lash RR, Harmon JR, et al. Serological investigation of heartland virus (Bunyaviridae: Phlebovirus) exposure in wild and domestic animals adjacent to human case sites in Missouri 2012-2013. Am J Trop Med Hyg. 2015;92:1163–7. 10.4269/ajtmh.14-0702 25870419 PMC4458820

[12] Clarke LL, Ruder MG, Mead DG, Howerth EW. Heartland virus exposure in white-tailed deer in the southeastern United States, 2001‒2015. Am J Trop Med Hyg. 2018;99:1346–9. 10.4269/ajtmh.18-0555 30255829 PMC6221220

[13] Riemersma KK, Komar N. Heartland virus neutralizing antibodies in vertebrate wildlife, United States, 2009‒2014. Emerg Infect Dis. 2015;21:1830–3. 10.3201/eid2110.150380 26401988 PMC4593439

[14] Raghavan RK, Peterson AT, Cobos ME, Ganta R, Foley D. Current and Future Distribution of the Lone Star Tick, *Amblyomma americanum* (L.) (Acari: Ixodidae) in North America. PLoS One. 2019;14:e0209082. 10.1371/journal.pone.0209082 30601855 PMC6314611

[15] Bosco-Lauth AM, Calvert AE, Root JJ, Gidlewski T, Bird BH, Bowen RA, et al. Vertebrate host susceptibility to Heartland virus. Emerg Infect Dis. 2016;22:2070–7. 10.3201/eid2212.160472 27869591 PMC5189141

[16] Clarke LL, Ruder MG, Mead D, Howerth EW. Experimental infection of white-tailed deer (*Odocoileus virginanus*) with Heartland virus. Am J Trop Med Hyg. 2018;98:1194–6. 10.4269/ajtmh.17-0963 29488458 PMC5928841

[17] Carlson AL, Pastula DM, Lambert AJ, Staples JE, Muehlenbachs A, Turabelidze G, et al. Heartland virus and hemophagocytic lymphohistiocytosis in immunocompromised patient, Missouri, USA. Emerg Infect Dis. 2018;24:893–7. 10.3201/eid2405.171802 29664369 PMC5938783

[18] Decker MD, Morton CT, Moncayo AC. One confirmed and 2 suspected cases of Heartland virus disease. Clin Infect Dis. 2020;71:3237–40. 10.1093/cid/ciaa647 32459327

[19] Muehlenbachs A, Fata CR, Lambert AJ, Paddock CD, Velez JO, Blau DM, et al. Heartland virus-associated death in tennessee. Clin Infect Dis. 2014;59:845–50. 10.1093/cid/ciu434 24917656 PMC4608028

[20] Staples JE, Pastula DM, Panella AJ, Rabe IB, Kosoy OI, Walker WL, et al. Investigation of Heartland virus disease throughout the United States, 2013‒2017. Open Forum Infect Dis. 2020;7:a125. 10.1093/ofid/ofaa125 32478118 PMC7246346

[21] Fill MA, Compton ML, McDonald EC, Moncayo AC, Dunn JR, Schaffner W, et al. Novel clinical and pathologic findings in a Heartland virus‒associated death. Clin Infect Dis. 2017;64:510–2. 27927857 10.1093/cid/ciw766 PMC5393941

[22] Kosoy OI, Lambert AJ, Hawkinson DJ, Pastula DM, Goldsmith CS, Hunt DC, et al. Novel thogotovirus associated with febrile illness and death, United States, 2014. Emerg Infect Dis. 2015;21:760–4. 10.3201/eid2105.150150 25899080 PMC4412252

[23] Savage HM, Burkhalter KL, Godsey MS Jr, Panella NA, Ashley DC, Nicholson WL, et al. Bourbon virus in field-collected ticks, Missouri, USA. Emerg Infect Dis. 2017;23:2017–22. 10.3201/eid2312.170532 29148395 PMC5708220

[24] Komar N, Hamby N, Palamar MB, Staples JE, Williams C. Indirect evidence of Bourbon virus (Thogotovirus, Orthomyxoviridae) infection in North Carolina. N C Med J. 2020;81:214–5. 10.18043/ncm.81.3.214 32366639

[25] Davidson WR, Siefken DA, Creekmore LH. Seasonal and annual abundance of *Amblyomma americanum* (Acari: Ixodidae) in central Georgia. J Med Entomol. 1994;31:67–71. 10.1093/jmedent/31.1.67 8158632

[26] Keirans JE, Litwak TR. Pictorial key to the adults of hard ticks, family Ixodidae (Ixodida: Ixodoidea), east of the Mississippi River. J Med Entomol. 1989;26:435–48. 10.1093/jmedent/26.5.435 2795615

[27] Keirans JE, Durden LA. Illustrated key to nymphs of the tick genus *Amblyomma* (Acari: Ixodidae) found in the United States. J Med Entomol. 1998;35:489–95. 10.1093/jmedent/35.4.489 9701933

[28] Paddock CD, Fournier P-E, Sumner JW, Goddard J, Elshenawy Y, Metcalfe MG, et al. Isolation of *Rickettsia parkeri* and identification of a novel spotted fever group *Rickettsia* sp. from Gulf Coast ticks (*Amblyomma maculatum*) in the United States. Appl Environ Microbiol. 2010;76:2689–96. 10.1128/AEM.02737-09 20208020 PMC2863434

[29] Lanciotti RS, Kerst AJ, Nasci RS, Godsey MS, Mitchell CJ, Savage HM, et al. Rapid detection of west nile virus from human clinical specimens, field-collected mosquitoes, and avian samples by a TaqMan reverse transcriptase-PCR assay. J Clin Microbiol. 2000;38:4066–71. 10.1128/JCM.38.11.4066-4071.2000 11060069 PMC87542

[30] Guindon S, Dufayard JF, Lefort V, Anisimova M, Hordijk W, Gascuel O. New algorithms and methods to estimate maximum-likelihood phylogenies: assessing the performance of PhyML 3.0. Syst Biol. 2010;59:307–21. 10.1093/sysbio/syq010 20525638

[31] Bacon RM, Pilgard MA, Johnson BJ, Piesman J, Biggerstaff BJ, Quintana M. Rapid detection methods and prevalence estimation for *Borrelia lonestari glpQ* in *Amblyomma americanum* (Acari: Ixodidae) pools of unequal size. Vector Borne Zoonotic Dis. 2005;5:146–56. 10.1089/vbz.2005.5.146 16011431

[32] Gleim ER, Garrison LE, Vello MS, Savage MY, Lopez G, Berghaus RD, et al. Factors associated with tick bites and pathogen prevalence in ticks parasitizing humans in Georgia, USA. Parasit Vectors. 2016;9:125. 10.1186/s13071-016-1408-6 26935205 PMC4776404

[33] Dobler G. Zoonotic tick-borne flaviviruses. Vet Microbiol. 2010;140:221–8. 10.1016/j.vetmic.2009.08.024 19765917

[34] Stefanoff P, Pfeffer M, Hellenbrand W, Rogalska J, Rühe F, Makówka A, et al. Virus detection in questing ticks is not a sensitive indicator for risk assessment of tick-borne encephalitis in humans. Zoonoses Public Health. 2013;60:215–26.https://www.ncbi.nlm.nih.gov/entrez/query.fcgi?cmd=Retrieve&db=PubMed&list_uids=22765504&dopt=Abstract 10.1111/j.1863-2378.2012.01517.x 22765504

[35] Brackney DE, Armstrong PM. Transmission and evolution of tick-borne viruses. Curr Opin Virol. 2016;21:67–74. 10.1016/j.coviro.2016.08.005 27569396

[36] Luo LM, Zhao L, Wen HL, Zhang ZT, Liu JW, Fang LZ, et al. *Haemaphysalis longicornis* ticks as reservoir and vector of severe fever with thrombocytopenia syndrome virus in China. Emerg Infect Dis. 2015;21:1770–6. 10.3201/eid2110.150126 26402039 PMC4593435

[37] Donaldson KA, Griffin DW, Paul JH. Detection, quantitation and identification of enteroviruses from surface waters and sponge tissue from the Florida Keys using real-time RT-PCR. Water Res. 2002;36:2505–14. 10.1016/S0043-1354(01)00479-1 12153016

[38] Shema Mugisha C, Vuong HR, Puray-Chavez M, Bailey AL, Fox JM, Chen RE, et al. A simplified quantitative real-time PCR assay for monitoring SARS-CoV-2 growth in cell culture. MSphere. 2020;5:e00658–20. 10.1128/mSphere.00658-20 32878932 PMC7471006

[39] Yun SM, Park SJ, Park SW, Choi W, Jeong HW, Choi YK, et al. Molecular genomic characterization of tick- and human-derived severe fever with thrombocytopenia syndrome virus isolates from South Korea. PLoS Negl Trop Dis. 2017;11:e0005893. 10.1371/journal.pntd.0005893 28937979 PMC5627960

[40] Egizi A, Bulaga-Seraphin L, Alt E, Bajwa WI, Bernick J, Bickerton M, et al. First glimpse into the origin and spread of the Asian longhorned tick, *Haemaphysalis longicornis*, in the United States. Zoonoses Public Health. 2020;67:637–50. 10.1111/zph.12743 32638553

