# Supplementary material for: Isolation of Heartland Virus from Lone Star Ticks, Georgia, USA, 2019
Source: Emerg Infect Dis. 2022 Apr;28(4):786–92. doi: 10.3201/eid2804.211540 (PMC8962890; doi:10.3201/eid2804.211540)
Supplement: Supplementary file 1 — Appendix. Additional information on isolation of Heartland virus from lone star ticks, Georgia¸ USA, 2019. [file 21-1540-Techapp-s1.pdf]

Article DOI: https://doi.org/10.3201/eid2804.211540

Isolation of Heartland Virus from Lone Star Ticks, Georgia, USA, 2019

Appendix

Appendix Table 1. Sequencing metrics for 3 genome segments from 3 Heartland virus–positive pools collected in Georgia, USA, 2019

Sample no.	Total reads	Small coverage,%	Mean small depth	Medium coverage, %	Mean medium depth	Large coverage, %	Mean large depth
HRT23	3,908,804	98.0	21	99.3	52	98.7	24
HRT26	4,625,523	95.7	24	100.0	76	99.2	50
HRT504	5,575,666	85.1	8	99.5	40	88.8	7

Appendix Table 2. Pairwise nucleotide identity between 3 HRTV virus genomes sequenced in this study, Georgia, 2019, and the only 3 previously published HRTV genomes*

		Small segment, % nucleotide identity					Medium segment, % nucleotide identity					Large segment, % nucleotide identity				
Sample	Location	HRT23	HRT26	HRT 504	JX00584X	KJ74014X	HRT23	HRT26	HRT 504	JX00584X	KJ74014X	HRT23	HRT26	HRT 504	JX00584X	KJ74014X
HRT23	Site 1	/					/					/				
HRT26	Site 1	100.0					100.0					99.8				
HRT504	Site 2	97.6†	97.6†				99.7	99.8				91.6†	91.8†			
JX00584X	Missouri 2009, human	97.5	97.4	95.1†			95.6	95.6	95.5			97.8	97.9	90.1†		
KJ74014X	Tennessee 2013, human	97.6	97.4	95.2†	97.4		98.4	98.4	98.2	95.7		98.0	98.1	90.4†	98.0	
NC_02449X	Missouri 2009, human	97.9	97.8	95.6†	98.3	97.9	98.2	98.2	98.1	95.5	98.4	97.9	98.0	90.2†	98.1	98.0

*HRTV, Heartland virus; / indicates that the percentage identity shown includes sequence ambiguities.
†For sample HRT504, only partial small and large segments were sequenced.

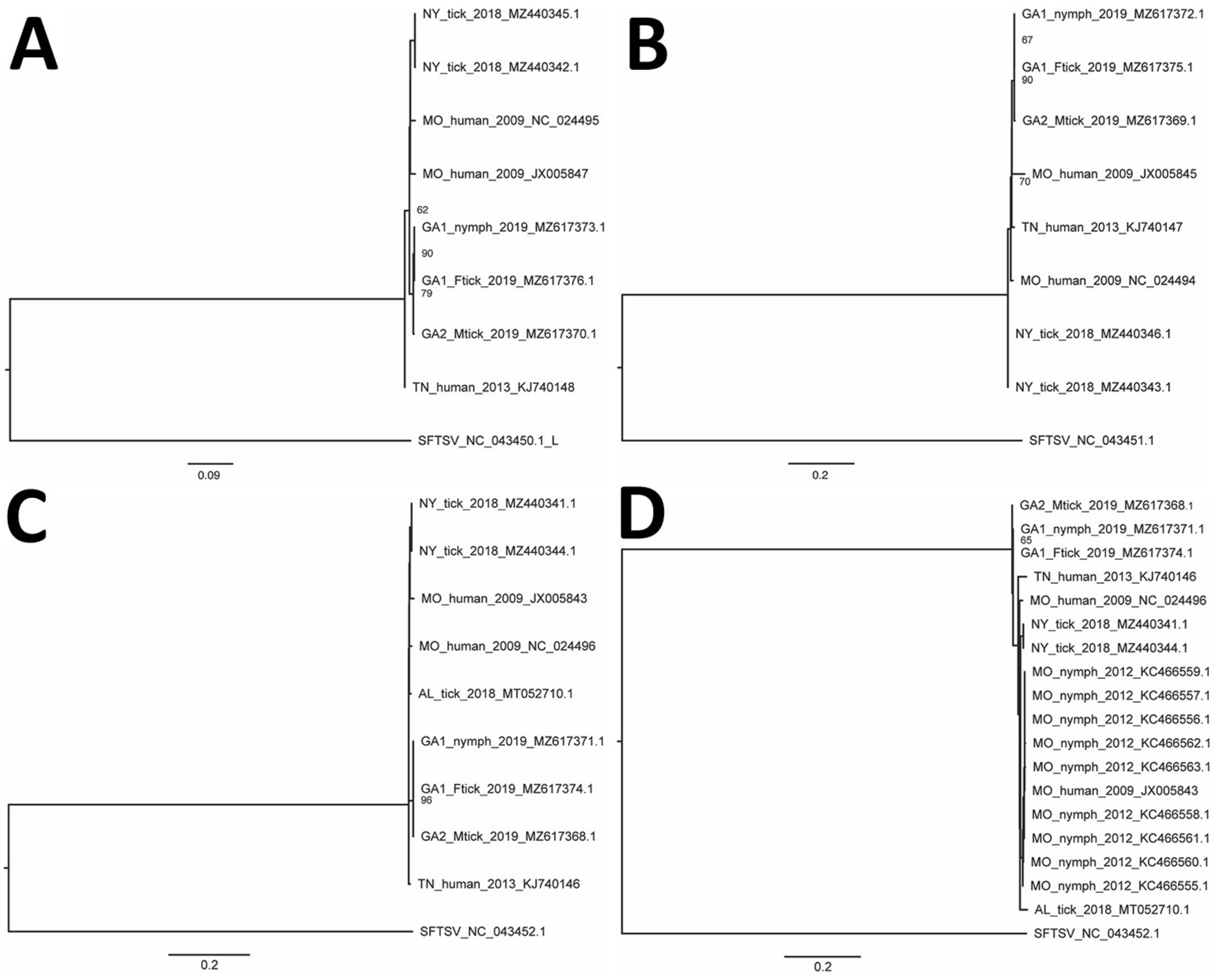
A
NY_tick_2018_MZ440345.1
NY_tick_2018_MZ440342.1
MO_human_2009_NC_024495
MO_human_2009_JX005847
62
GA1_nymph_2019_MZ617373.1
90
GA1_Ftick_2019_MZ617376.1
79
GA2_Mtick_2019_MZ617370.1
TN_human_2013_KJ740148
SFTSV_NC_043450.1_L
0.09
B
GA1_nymph_2019_MZ617372.1
67
GA1_Ftick_2019_MZ617375.1
90
GA2_Mtick_2019_MZ617369.1
MO_human_2009_JX005845
70
TN_human_2013_KJ740147
MO_human_2009_NC_024494
NY_tick_2018_MZ440346.1
NY_tick_2018_MZ440343.1
SFTSV_NC_043451.1
0.2
C
NY_tick_2018_MZ440341.1
NY_tick_2018_MZ440344.1
MO_human_2009_JX005843
MO_human_2009_NC_024496
AL_tick_2018_MT052710.1
GA1_nymph_2019_MZ617371.1
GA1_Ftick_2019_MZ617374.1
96
GA2_Mtick_2019_MZ617368.1
TN_human_2013_KJ740146
SFTSV_NC_043452.1
0.2
D
GA2_Mtick_2019_MZ617368.1
GA1_nymph_2019_MZ617371.1
65
GA1_Ftick_2019_MZ617374.1
TN_human_2013_KJ740146
MO_human_2009_NC_024496
NY_tick_2018_MZ440341.1
NY_tick_2018_MZ440344.1
MO_nymph_2012_KC466559.1
MO_nymph_2012_KC466557.1
MO_nymph_2012_KC466556.1
MO_nymph_2012_KC466562.1
MO_nymph_2012_KC466563.1
MO_human_2009_JX005843
MO_nymph_2012_KC466558.1
MO_nymph_2012_KC466561.1
MO_nymph_2012_KC466560.1
MO_nymph_2012_KC466555.1
AL_tick_2018_MT052710.1
SFTSV_NC_043452.1
0.2
